# Endothelial progenitor cells promote viability and nerve regenerative ability of mesenchymal stem cells through PDGF-BB/PDGFR-β signaling

**DOI:** 10.18632/aging.102604

**Published:** 2020-01-03

**Authors:** Jiafeng Fang, Xuna Huang, Xiaoyan Han, Zongheng Zheng, Cheng Hu, Tufeng Chen, Xiaofeng Yang, Xi Ouyang, Zehong Chen, Hongbo Wei

**Affiliations:** 1 Department of Gastrointestinal Surgery, The Third Affiliated Hospital of Sun Yat-sen University, Guangzhou 510630, China; 2 Central Laboratory, The Third Affiliated Hospital of Sun Yat-sen University, Guangzhou 510630, China; 3 Department of Urinary Surgery, The Third Affiliated Hospital of Sun Yat-sen University, Guangzhou 510630, China

**Keywords:** cell viability, endothelial progenitor cells, erectile dysfunction, mesenchymal stem cells, platelet-derived growth factor

## Abstract

Denervation-induced erectile dysfunction (ED) is a prevailing health problem. Our previous study revealed that endothelial progenitor cells (EPCs) promoted the effect of mesenchymal stem cells (MSCs) on restoration of denervation-induced ED in rats. However, underling mechanisms are still largely elusive. In this study, EPCs and MSCs were co-cultured and resorted to co-EPCs and co-MSCs. EPCs-derived paracrine factors containing PDGF-BB (platelet-derived growth factor) were detected, and MSCs were pre-treated with PDGF-BB, while co-MSCs were pre-treated with PDGFR inhibitor AG1296. Either viability or nerve regenerative ability of MSCs was evaluated. In addition, inhibition of either PI3K/Akt or MEK/Erk pathway was performed to evaluate the role of PI3K/Akt and MEK/Erk pathway in PDGF-BB-induced viability of MSCs. The results revealed that PDGF-BB significantly increased the proportion of PDGFR-β^+^ MSCs, and promoted both *in-vitro* and *in-vivo* viability, as well as nerve regenerative capacity and erectile function restoration of MSCs in rats. Inhibition of PI3K/Akt, MEK/Erk pathway or mTOR led to decrease of PDGF-BB/PDGFR-β induced viability of MSCs. To our knowledge, our study first demonstrates that EPCs promote viability and potential nerve regenerative ability of MSCs through PDGF-BB/PDGFR-β signaling and its downstream PI3K/Akt and MEK/Erk pathways. mTOR acts as a co-mediator in PI3K/Akt and MEK/Erk pathways.

## INTRODUCTION

Due to intraoperative damage of pelvic autonomic nerve, erectile dysfunction (ED) frequently occurs after pelvic surgery [1, 2]. Unfortunately, traditional treatment is less effective for denervation-induced ED treatment [3]. Previous studies have documented that the adoptive transfer of mesenchymal stem cells (MSCs) is effective for ED in animals [4, 5]. Our previous study also proved that periprostatic implantation of neuronal differentiated MSCs was effective on ameliorating erectile function in rats. The mechanism may be ascribed to both decrease of apoptotic cells and paracrine effect of MSCs [6]. However, a clinical trial of stem cell therapy for ED revealed that despite having increased penile rigidity, none of the 7 diabetic related ED patients was able to achieve vaginal penetration [7]. The lack of efficacy could be ascribed to multiple factors, containing insufficient understanding of pharmacokinetics of the administered cells. Thus, more efficient studies on the use of MSCs therapy are in need to restore erectile function better.

Endothelial progenitor cells (EPCs) are mononuclear cells circulating in the blood and participating in vascular repair. Since firstly discovered in 1997, EPCs have been of great interest due to its potential to differentiate into capillary endothelial cells and play a supportive role in angiogenesis vascular repair [8]. EPCs may be isolated from bone marrow, adult peripheral blood, umbilical cord blood and human-induced pluripotent stem cells. However, only EPCs derived from adult peripheral blood and umbilical cord blood are considered as pure EPCs, and have advantages of noninvasive isolation and low immunogenicity [9, 10]. Recently, EPCs have been widely applied in co-culturing with other cells, serving as a source of angiocrine factors [11]. Previous multiple research studies revealed that combined transplantation of MSCs and EPCs is more effective than single-cell transplantation for cardiovascular disease [12], cerebrovascular disease [13] and bone-related disease [14]. In our previous study, we also demonstrated that combined transplantation of MSCs and EPCs resulted in a better effect than either cell transplantation alone on restoration of erectile function [15].

Nonetheless, how EPCs exert enhanced function in support of MSCs and restoration of denervation-induced ED is still largely elusive. Recent study revealed that EPCs may promote MSCs engraftment via platelet-derived growth factor (PDGF-BB)/PDGFR-β signaling [16]. Other studies also revealed the function of PDGF-BB/PDGFR-β signaling in MSCs viability [17, 18]. In this study, we investigated the PDGF-BB/PDGFR-β signaling and its downstream pathway in enhancement of viability and nerve regeneration ability of MSCs *in vitro* and *in vivo* denervation-induced ED model.

## RESULTS

### Co-culture of MSCs and EPCs decrease expression level of PDGF-BB and increases proportion of PDGFR-β^+^ MSCs

**Figure 1:**
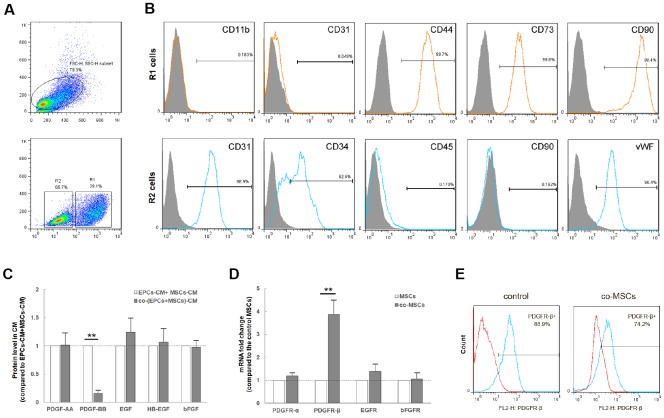
**Co-culture of MSCs and EPCs decreased level of PDGF-BB and increases proportion of PDGFR-β^+^ MSCs.** The co-culture cells were sorted into co-MSCs (R1, CD90^+^) and co-EPCs (R2, CD90^-^) by FACS (**A**). Flow cytometry revealed that R1 cells expressed the mesenchymal stem cell markers CD44, CD73 and CD90, but not hematopoietic or endothelial markers CD11b and CD31. R2 cells expressed hematopoietic and endothelial markers CD31, CD34 and vWF, but not mesenchymal stem cell markers CD45 and CD90. Real-time PCR revealed that after co-culture, the expression level of PDGF-BB in co-EPCs significantly decreased. Results are mean ± SD from three independent experiments (**C**). Correspondingly, the expression level of PDGFR-β in co-MSCs increased obviously. Results are mean ± SD from three independent experiments (**D**). In addition, flow cytometry revealed that detection of PDGFR-β decreased in co-MSCs due to combination of PDGF-BB and PDGFR-β (**E**). **P<0.01.

After 72h co-culture, the mixed cells were sorted into co-MSCs and co-EPCs by FACS (Figure 1A). The sorted cells were further identified to be MSCs and EPCs by flow cytometry (Figure 1B). In order to figure out which EPCs-derived trophic factor was related to MSCs viability, factors secreted only by EPCs but not by MSCs, containing PDGF-AA, PDGF-BB, EGF, HB-EGF and bFGF were detected. As shown in Figure 1C, compared with that of EPCs and MSCs, the level of PDGF-BB in co-cells significantly decreased. Accordingly, compared with that of MSCs, the level of PDGFR-β in co-MSCs increased (Figure 1D), and detection of PDGFR-β decreased (Figure 1E), suggesting preferential utilization of EPCs-derived PDGF-BB by co-MSCs.

### EPCs enhance *in-vitro* viability of MSCs through PDGF-BB/ PDGFR-β signaling

**Figure 2:**
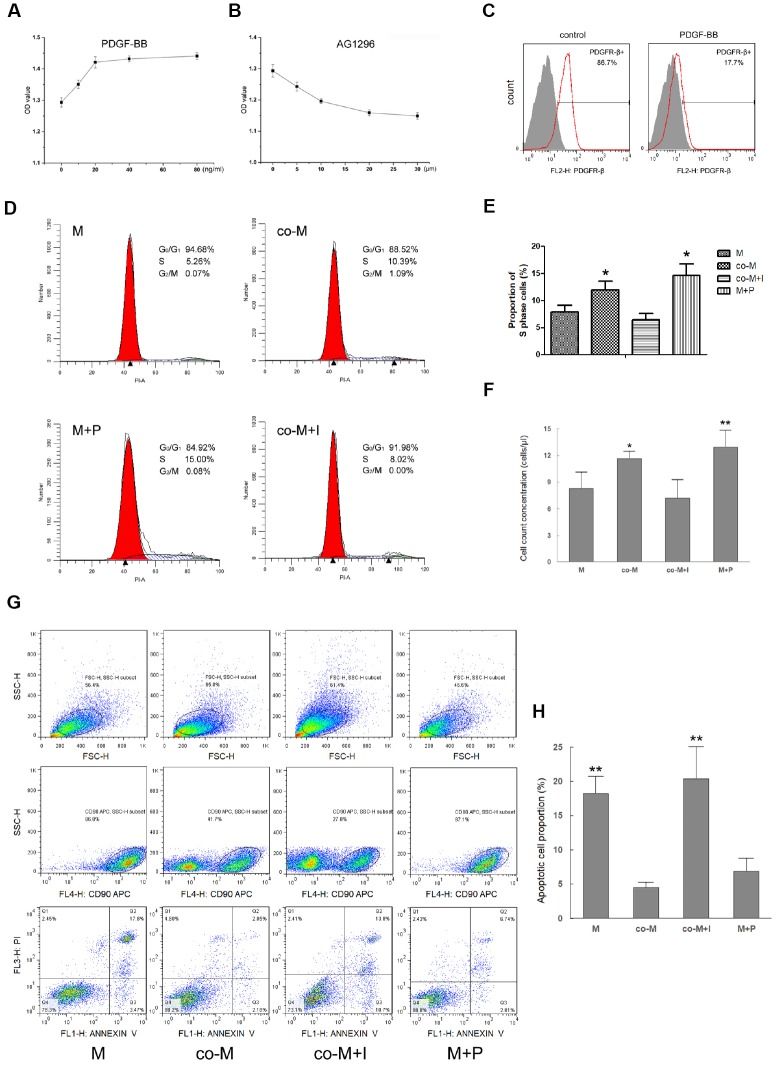
**Effect of PDGF-BB/PDGFR-β signaling on viability of MSCs.** MSCs were treated with PDGF-BB at concentrations of 10, 20, 40 or 80ng/ml. CCK-8 assay revealed maximal proliferation of MSCs induced with PDGF-BB at concentration of 20 ng/ml or more. Results are mean ± SD from three independent experiments (**A**). co-MSCs were treated with AG1296 (PDGFR-β inhibitor), and CCK-8 assay revealed maximal inhibition of proliferation at concentration of 20μm or more. Results are mean ± SD from three independent experiments (**B**). Flow cytometry revealed that after treated with 20ng/ml PDGF-BB, detection of PDGFR-β^+^ MSCs decreased due to combination of PDGF-BB and PDGFR-β (**C**). The cell cycle analysis noted that compared with M and co-M+I groups, the proportion of cells in S phase significantly increased in either co-M or M+P group. Results are mean ± SD from three independent experiments (**D**, **E**). Cell count concentration was assessed using the CountBright with flow cytometry. The result was consistent with the cell cycle. Results are mean ± SD from three independent experiments (**F**). Cell apoptosis detected with flow cytometry revealed that compared with M and co-M+I groups, the proportion of apoptotic cells significantly decreased in either co-M or M+P group (**G**, **H**). *P<0.05 vs. M or co-M+I group, **P<0.01 vs. M or co-M+I group.

To assess the effect of PDGF-BB/PDGFR-β signaling on EPCs induced viability of MSCs, MSCs were pre-treated with PDGF-BB (M+P group) or without PDGF-BB (M group), while co-MSCs were pre-treated with PDGFR inhibitor AG1296 (co-M+I group) or without AG1296 (co-M group). The maximal effect was observed with PDGF-BB or AG1296 of more than 20ng/ml or 20μm (Figure 2A, 2B). Flow cytometry revealed that 20ng/ml PDGF-BB significantly bound with PDGFR-β and thus decreased detection of PDGFR-β (Figure 2C). The cell cycle analysis revealed that compared with M (7.90±1.21) and co-M+I (6.40±1.18) groups, the proportion of cells in S phase significantly increased in either co-M (12.00±1.58) or M+P (14.67±2.07) group (Figure 2D, 2E). Additionally, the result of cell count concentration revealed that compared with M (8.30±1.82) and co-M+I group (7.22±2.04), cell count concentrations of co-M (11.62±0.85) and M+P group (12.92±1.91) increased significantly (Figure 2F). Cell apoptosis assay revealed that compared with M (18.17±2.52) and co-M+I (20.33±5.59) groups, the proportion of apoptotic cells significantly decreased in either co-M (4.51±0.70) or M+P (6.91±1.81) group (Figure 2G, 2H).

**Figure 3:**
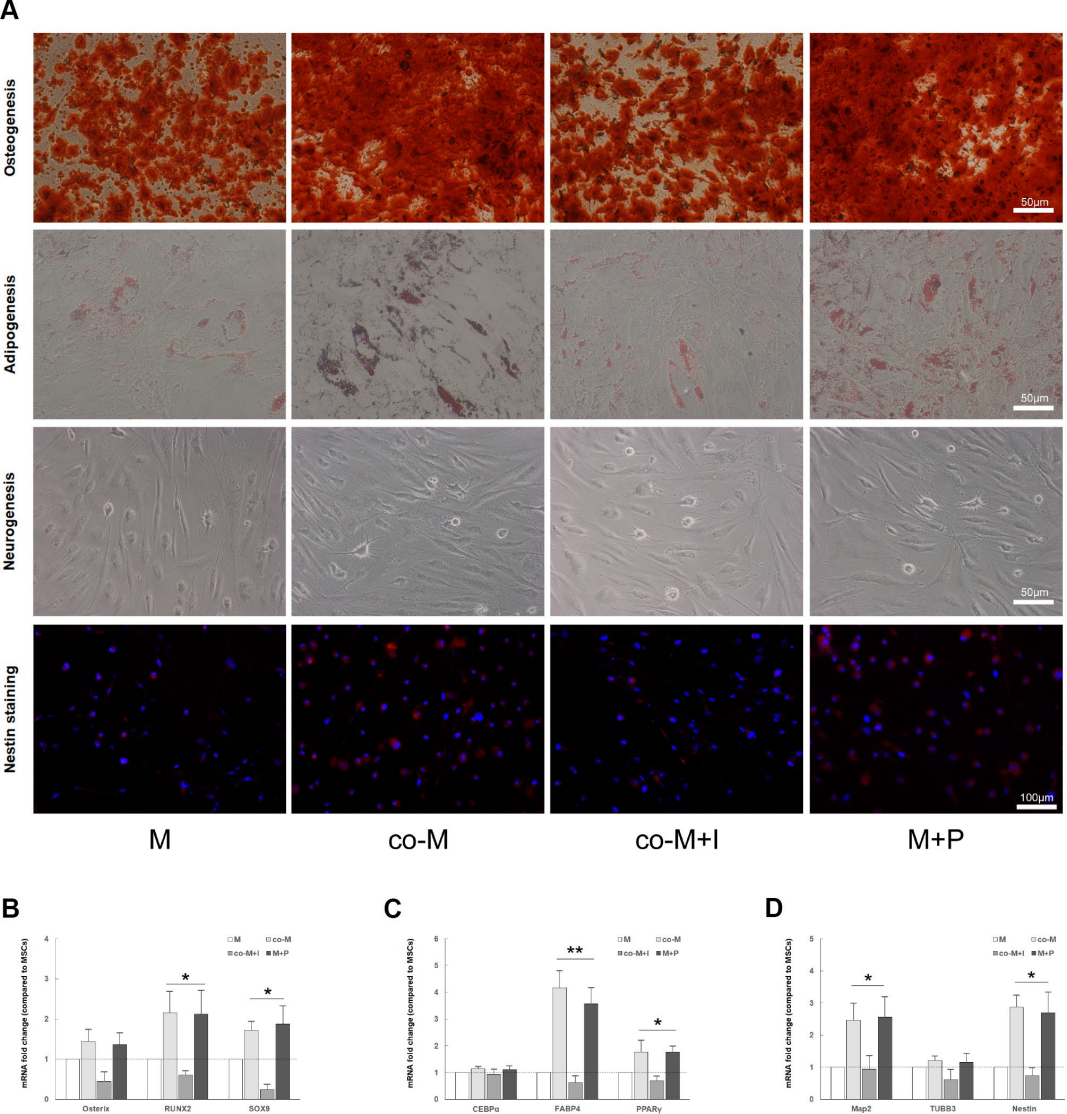
***In vitro* multilineage differentiation of MSCs induced with PDGF-BB/PDGFR-β signaling.** Cells were cultured and induced in the osteogenic, adipogenic or neural differentiation medium, respectively. The staining result revealed that the osteogenic (alizarin red S), adipogenic (oil red O) or neural (Nestin) differentiation capacity in co-M and M+P groups was better than that of M or co-M+I group (**A**). The RNA of adipogenic, osteogenic or neural markers were extracted and detected. Compared with the M and co-M+I group, the expression levels of osteogenic (RUNX2 and SOX9), adipogenic (FABP4 and PPARγ) and neural (MAP2 and Nestin) markers significantly increased in co-M and M+P groups. Results are mean ± SD from three independent experiments (**B**–**D**). *P<0.05 vs. M or co-M+I group, **P<0.01 vs. M or co-M+I group.

To assess the *in vitro* multilineage differentiation, cells were cultured and induced in the osteogenic, adipogenic or neural differentiation medium, respectively. The staining result revealed that the multilineage differentiation capacity in co-M and M+P groups was significantly stronger than M or co-M+I group (Figure 3A). Real-time PCR revealed that the expression levels of adipogenic (FABP4 and PPARγ), osteogenic (RUNX2 and SOX9) and neural (MAP2 and Nestin) markers significantly increased in co-M and M+P groups (Figure 3B–3D).

### EPCs enhance *in vivo* viability of MSCs through PDGF-BB/ PDGFR-β signaling

**Figure 4:**
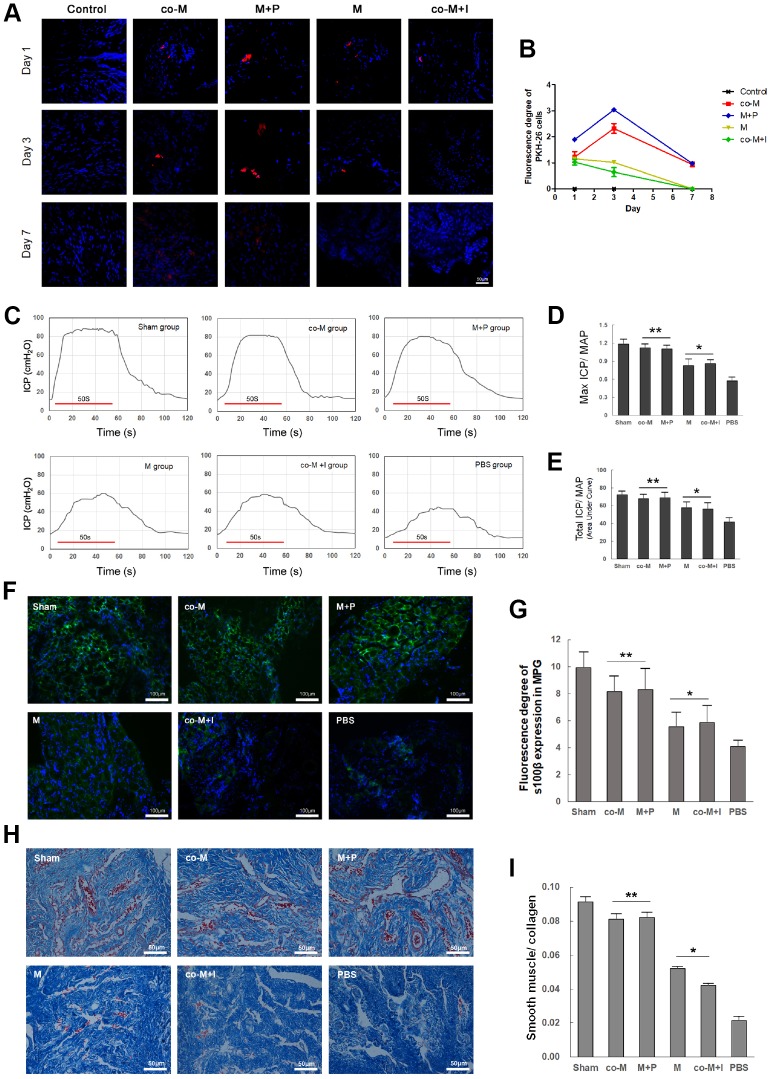
***In vivo* viability and nerve regenerative capacity of MSCs induced with PDGF-BB/PDGFR-β signaling. PKH26-labled cells (Red) were detected in the MPG and cavernous nerve 1, 3, 7 and 14 days after implantation.** PKH26-labled cells were detectable within 3 days after implantation in either M or co-M+I group. However, they were durable and detectable until 7 days after implantation in both co-M and M+P groups. They were undetectable 14 days after implantation in all groups (data not shown). Image J revealed a stronger fluorescence degree of PKH-26 cells in the co-M and M+P groups than either M or co-M+I group (**B**). Rats per group: n=2. Scale bar=50μm. Transplantation of different groups of MSCs restored erectile function in CNI rats (**C**–**E**). Representative ICP responses for the sham, M, co-M, co-M+I, M+P and PBS group 2 weeks after treatment of stem cells (**C**). Compared with the PBS group, treatment of MSCs significantly increased the mICP/MAP and tICP/MAP ratios. Moreover, the data of the co-M and M+P group were better than the M or co-M+I group (**D**, **E**). Each bar represents mean ± SD (n=5 animals per group). *P<0.05 vs. PBS group; **P<0.01 vs. PBS group and *P*<0.05 vs. M and co-M+I group. MAP=mean arterial pressure. The expressions of neural marker S100β in MPG were detected 2 weeks after treatment of stem cells (**F**). The ratio of smooth muscle to collagen in penis was assessed with Masson’s trichrome staining (**H**). Quantitative analysis was performed using Image J (**G**, **I**). Each bar represented means ± SD (n=5 animals per group). *P<0.05 vs. PBS group; **P<0.01 vs. PBS group and *P*<0.05 vs. M and co-M+I group.

To determine the *in vivo* viability of implanted MSCs, PKH26-labeled cells were detected in the MPG and cavernous nerve 1, 3, 7 and 14 days after implantation. As shown in Figure 4A, PKH26-labeled cells were only detectable within 3 days after implantation in either M or co-M+I group. However, PKH26-labeled cells were durable and detectable until 7 days after implantation in both co-M and M+P groups. PKH26-labeled cells were undetectable 14 days after implantation in all groups (data not shown). The fluorescence degree of PKH-26 cells was evaluated with Image J (Figure 4B).

### EPCs promote nerve regenerative capacity and erectile function restoration of MSCs through PDGF-BB/ PDGFR-β signaling

Erectile function was evaluated by electrical stimulation of the CN 2 weeks after CNI and treatment. There were no significant differences in the MAP among different groups (data not shown). Compared with the PBS group (0.57±0.06 and 41.64±4.54), the mICP/MAP and tICP/MAP ratios in M (0.83±0.11 and 57.60±6.37), co-M (1.12±0.06 and 67.81±4.62), co-M+I (0.86±0.07 and 56.21±6.79) and M+P group (1.10±0.06 and 68.80±5.88) significantly increased. Furthermore, the data of the co-M and M+P group were better than the M or co-M+I group (Figure 4C–4E).

The MPG segments were examined for the neural marker S100β by immunofluorescence staining 2 weeks after MSCs implantation. As shown in Figure 4F, transplantations of MSCs restored the expression of S100β in CNI rats. Compared with implantation of MSCs or co-M+I cells, transplantation of co-MSCs or M+P cells exhibited higher expression of S100β (Figure 4G).

The penis segments were used to assess the ratio of smooth muscle to collagen with Masson’s trichrome staining. As shown in Figure 4H, transplantations of MSCs restored the ratio of smooth muscle to collagen in CNI rats. Compared with implantation of MSCs or co-M+I cells, transplantation of co-MSCs or M+P cells exhibited higher ratios (Figure 4I).

### The role of PI3K/Akt and MEK/Erk pathway in PDGF-BB-induced viability of MSCs

**Figure 5:**
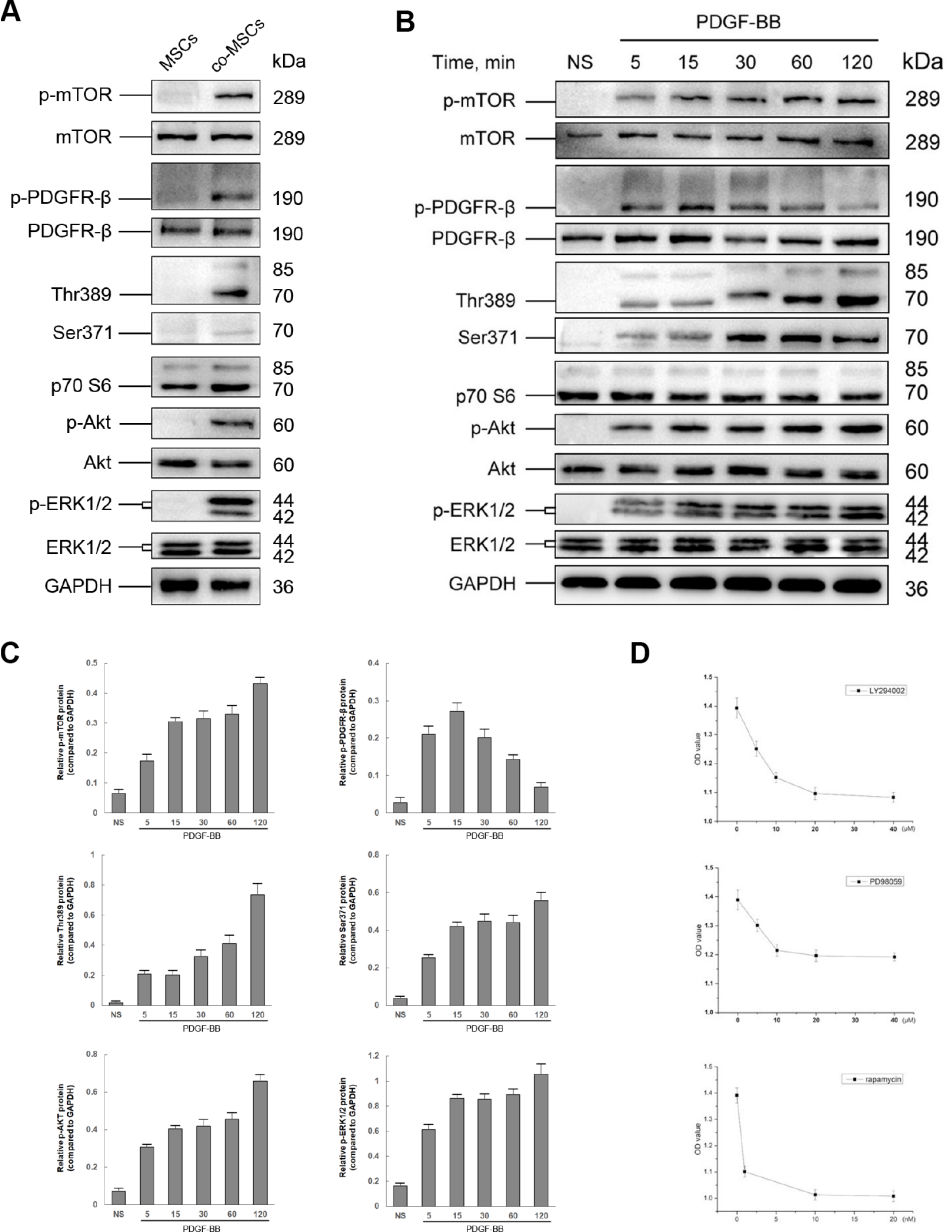
**The role of PI3K/Akt and MEK/Erk pathway in PDGF-BB-induced viability of MSCs.** Either co-cultured with EPCs or 20 ng/ml PDGF-BB was applied to induced viability of MSCs and the status in phosphorylation of both PI3K/Akt and MEK/Erk pathways was assessed by western blot. Co-cultured with EPCs, the co-MSCs revealed phosphorylation of PDGFR-β, Akt, mTOR, p70 S6 Kinase (Thr389 and Ser371) and ERK ½ (**A**). Similarly, after induced with PDGF-BB, PDGFR-β, Akt, mTOR, p70 S6 Kinase and ERK ½ was rapidly phosphorylated (**B**). The densitometry of western blots was evaluated by Image J and the results revealed that phosphorylation of PDGFR-β reached peak at 15 min and gradually returned to basal level. However, the phosphorylation of downstream pathway factors consistently existed. Results are mean ± SD from three independent experiments (**C**). Different concentrations of LY294002 (PI3K inhibitor), PD98059 (Erk inhibitor) or rapamycin (mTOR inhibitor) were pre-treated to MSCs 1h before treated with PDGF-BB. CCK-8 cell proliferation test revealed that the optimal concentration of LY294002, PD98059 and rapamycin was 20μM, 10μM and 10nM, respectively. Results are mean ± SD from three independent experiments (**D**).

To investigate the downstream pathways involved in PDGF-BB induced viability of MSCs, phosphorylation of PDGFR-β, Akt, ERK1/2, mTOR and p70 S6 Kinase (Thr389 and Ser371) was assessed. After co-culture with EPCs (Figure 5A) or induced by 20ng/ml PDGF-BB (Figure 5B), PDGFR-β, Akt and ERK ½ were rapidly phosphorylated. Phosphorylation of PDGFR-β reached peak at 15 min and gradually returned to basal level. However, the phosphorylation of Akt and ERK ½ consistently existed. A similar consistent phosphorylation was observed for mTOR and p70 S6 Kinase (Figure 5C).

To determine the role of PI3K/Akt and MEK/Erk pathway in PDGF-BB-induced viability of MSCs, MSCs treated with PDGF-BB were pre-treated with PI3K inhibitor LY294002, Erk inhibitor PD98059 or mTOR inhibitor rapamycin, respectively. CCK-8 cell proliferation test was applied to determine the optimal concentration. As shown in Figure 5D, LY294002, PD98059 and rapamycin all inhibited MSCs viability induced with PDGF-BB, and the optimal concentration was 20μM, 10μM and 10nM, respectively.

**Figure 6:**
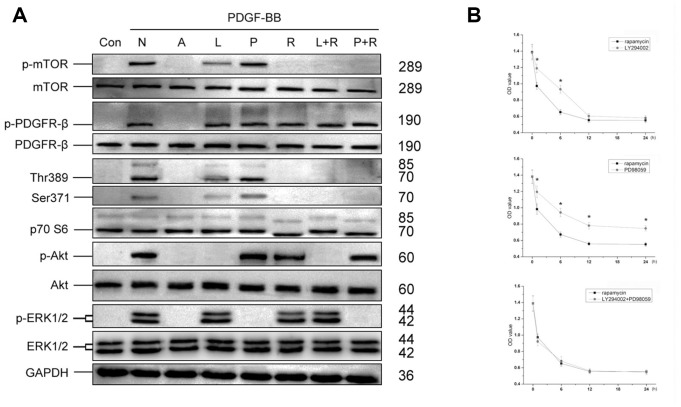
**The role of mTOR in PI3K/Akt and MEK/Erk pathways.** Optimal concentration of LY294002, PD98059 or rapamycin was added into MSCs culture 1h before treated with PDGF-BB. LY294002 significantly inhibited PDGF-BB induced phosphorylation of Akt, and partly inhibited phosphorylation of mTOR, Thr389 and Ser371, while PD98059 inhibited phosphorylation of ERK1/2. In contrast, rapamycin, rapamycin combined with LY294002 (L+R) or rapamycin combined with PD98059 (P+R) could completely inhibit phosphorylation of mTOR, Thr389 and Ser371 (**A**). Different inhibitors were added 1, 6, 12 or 24h before treated with PDGF-BB. The CCK-8 test revealed that compared with LY294002 or PD98059, rapamycin revealed better Inhibited effect of PDGF-BB-induced viability of MSCs, while combination of LY294002 and PD98059 revealed similar effect with rapamycin. Results are mean ± SD from three independent experiments (**B**). Con: Control; N: None; A: AG1296; L: LY294002; P: PD98059; R: rapamycin; L+R: LY294002+ rapamycin; P+R: PD98059+ rapamycin.

20μM LY294002, 10μM PD98059 or 10nM rapamycin was then added 1h before treated with PDGF-BB, and phosphorylation of PI3K/Akt and MEK/Erk pathways was assessed. As shown in Figure 6A, LY294002 significantly inhibited PDGF-BB induced phosphorylation of Akt, and partly inhibited phosphorylation of mTOR, Thr389 and Ser371, while PD98059 inhibited phosphorylation of ERK1/2. In contrast, combination of rapamycin with LY294002 or PD98059 almost completely inhibited phosphorylation of mTOR, Thr389 and Ser371. Thus, mTOR may act as a co-mediator of PI3K/Akt and MEK/Erk pathways in PDGF-BB-induced viability of MSCs. CCK-8 cell proliferation test proved that rapamycin had similar effect with combination of LY294002 and PD98059 on inhibiting PDGF-BB-induced viability of MSCs, while greater than single application of LY294002 or PD98059 (Figure 6B).

## DISCUSSION

Erectile dysfunction is a prevailing health problem that has serious influence on the quality of life of either men or their partners. Recently, stem cell therapy for ED has been widely studied [19]. However, the clinical efficiency of stem cells therapy for ED does not meet the expectation, which indicates an urgent need in finding more effective stem cells and methods [7]. With the deepening of research, more attentions have been paid on combination of cell types, such as MSCs and EPCs, in regenerative medicine [12–14]. EPCs have been proved to participate in angiogenesis and microvascular neovascularization, as well as serve as a trophic mediator for MSCs engraftment via paracrine signaling. Correspondingly, MSCs have strong ability to differentiate into specific injury cells and repair impaired tissues. Thus, It is expected that combined transplantation of the two types of cells should compensate for the limitations of transplantation of either MSCs or EPCs alone [12, 20]. Our previous study presented that compared with delivery of single cell, combined transplantation of MSCs and EPCs was more effective in restoration of erectile function of CNI rats [15]. In this study, we provided a more evidence that either co-MSCs or MSCs treated with PDGF-BB exhibited better viability both *in vitro* and *in vivo*, as well as greater effect on nerve regeneration and restoration of erectile function.

The exact mechanisms of EPCs-induced viability of MSCs are still elusive. More and more research studies revealed that EPCs and ECs may enhance tissue regeneration effect through secreting of paracrine factors, containing PDGF-AA, PDGF-BB, EGF, HB-EGF, bFGF, etc [21–23]. Further research found that among kinds of EPCs-derived factors, PDGF-BB played an important role in enhancing viability of MSCs [16, 18, 24]. PDGF-BB can bind and activate its receptor PDGFR-β in MSCs, thus increase proportion of PDGFR-β^+^ MSCs, which has been proved to have stronger stemness-related properties [17, 25]. In this study, we assessed concentrations of EPCs-derived trophic factors, containing PDGF-AA, PDGF-BB, EGF, HB-EGF and bFGF, in culture medium of EPCs, MSCs or co-culture cells using ELISA kits. The results revealed that after co-culture, only the secretion level of PDGF-BB obviously decreased and the ratio of PDGFR-β^+^ MSCs increased, suggesting preferential utilization of PDGF-BB by MSCs. Simultaneously, additional PDGF-BB or PDGFR-β inhibitor had obvious effect on viability of MSCs, implicating the important role of PDGF-BB/PDGFR-β signaling.

Previous studies have revealed a positive effect of PDGF on self-renewal, proliferation and stemness function of MSCs. However, whether PDGF promotes or inhibits the differentiation of MSCs is still controversial. Some studies demonstrated a negative effect of PDGF on differentiation of MSCs, but positive effect of proliferation [26, 27]. In this study, after co-cultured with EPCs, MSCs revealed better *in vitro* multilineage differentiation abilities, suggesting a positive effect of PDGF on differentiation of MSCs, which was consistent with some studies [28, 29]. That may because in this study, the MSCs differentiation is induced under specific medium. We supposed that PDGF-BB may enhance the viability of MSCs and then, the MSCs with better viability may have stronger differentiation ability under induced condition.

Although PDGF-BB has been implicated in the regulation of biological behavior of MSCs, the underlying signal transduction pathways remain largely controversial. Some research studies revealed that PDGF-BB enhanced cell viability through PI3k/Akt pathway [30, 31]. However, other studies demonstrated the importance of MEK/Erk pathway in PDGF-induced cell viability [26, 32]. Some experts believed that PI3k/Akt pathway was involved with proliferation of MSCs induced by PDGF-BB, and MEK/Erk pathway was related to differentiation of MSCs [27]. Other studies also indicated potential cross-talk and compensatory signaling between the two pathways [33–35]. Activated ERK signaling has been shown to activate mTOR and thus stimulate cell growth [33, 36]. However, whether the situation is similar in MSCs is unknown. In this study, we revealed that inhibition of either PI3k/Akt or MEK/Erk pathway could reverse PDGF-BB induced viability of MSCs, while the effect was more obvious for the former pathway. To further discuss the possibility of cross-talk between the two pathways, we inhibited mTOR, which was considered as a co-mediator between the PI3K and MEK pathways and an important target element for maximal PI3K pathway inhibition [33]. The effect of inhibition of mTOR was similar with combined inhibition of PI3k and MEK pathways. These results suggest that MEK/Erk pathway could at least partly promote the viability of MSCs through activating mTOR.

Some studies considered that MSCs repair damaged tissues through differentiating into specific cell types [37, 38]. However, in recent years, more and more evidences revealed that paracrine activity of MSCs played a key role in regenerative medicine [39, 40]. Our previous study also demonstrated that the expression levels of neurotrophic factors in co-culture MSCs/EPCs were significantly higher than those of mono-culture cells [15]. Taking together, we consider that EPCs promoted viability of MSCs through PDGF-BB/PDGFR-β signaling and downstream of PI3k/Akt and MEK/Erk pathways, and thus enhanced paracrine activity and nerve regeneration ability of MSCs.

There are some limitations in the present study. First, although the rats are the most commonly used animal in ED research, the rat CNI-ED model may not be equal to human beings. Thus, potential mammal ED model may be performed to reduce the limitation of rats in ED research in the future. Second, promotion of viability and proliferation of MSCs may be dangerous for patients who undergo radical prostatectomy or proctectomy. Although MSCs were undetectable 14 days after implantation in this study, suggesting that MSCs did not proliferate and survive without limit, more long-term observation should still be performed. Third, the doses of delivered stem cells and frequency of transplantation should be further investigated to find an optimal therapeutic method for nerve-injury-related ED. At last, either gene silence or overexpress of PDGFR-β should be performed in the future, to more intuitively verify the role of PDGFR-β and its downstream pathways in promoting the viability and potential nerve regenerative ability of MSCs.

In conclusion, to our knowledge, our study first reveals the role of PDGF-BB/PDGFR-β signaling, as well as downstream PI3K/Akt and MEK/Erk pathways, in promoting the viability and restoration of denervation-induced ED of MSCs. In addition, mTOR has been shown to be a co-mediator in PI3K/Akt and MEK/Erk pathways.

## MATERIALS AND METHODS

### Co-culture and fluorescence-activated cell sorting (FACS) of MSCs and EPCs

Institutional review board approval was obtained for all procedures. Written informed consent was obtained from the donors of the human bone marrow and umbilical cord blood samples. Human bone marrow MSCs and human umbilical cord blood EPCs were Isolated and cultured as previously described [15]. In addition, MSCs and EPCs were characterized using flow cytometry. Briefly, MSCs and EPCs were incubated with fluorescein isothiocyanate-conjugated antibodies for 30 minutes at 4ºC. The following antibodies were used (eBioscience, USA): CD11b, CD31, CD44, CD73 and CD90 for MSCs, CD31, CD34, CD45, CD90 and vWF for EPCs. The cells were washed twice with PBS. FACSCalibur flow cytometer and Cellquest software (BD Bioscience) were used to analyze antibody binding.

MSCs and EPCs at passage 3 were mixed at a ratio of 1:2. Cells were cultured in EBM-2, 5% FBS for 72h. After that, the mixed cells were prepared into single-cell suspensions, stained with FITC-conjugated anti-human CD90 antibody (eBioscience, USA), sorted into CD90^+^ (co-MSCs) and CD90^-^ (co-EPCs) cells by fluorescence-activated cell sorting (FACS). Sorted cells were analyzed either immediately or after an indicated period of culture. For flow cytometry, MSCs were incubated with fluorescein isothiocyanate-conjugated PDGFR-β antibody (eBioscience, USA) for 30 minutes at 4ºC. The cells were washed twice with phosphate-buffered saline (PBS). FACSCalibur flow cytometer and Cellquest software were used to analyze antibody binding.

### Enzyme-linked immunosorbent assay (ELISA)

The concentrations of EPCs-derived trophic factors in culture medium of EPCs, MSCs or co-culture cells were measured using ELISA kits (Quantikine ELISA kit, R&D Systems, Germany) according to the manufacturers’ instructions. The following factors were detected: PDGF-AA (Catalog #: DAA00B), PDGF-BB (Catalog #: DBB00), EGF (Catalog #: DEG00), HB-EGF (Catalog #: DHBEG0) and bFGF (Catalog #: DFB50). Taking detection of PDGF-BB for example. Briefly, 100 μL of assay diluent was added to each well of a 96-well plate, accompany with 100 μL of standard, control or sample to each well. Then the plate was covered with a plate sealer and incubated at room temperature for 2 hours. After washing for 4 times, 200 μL of conjugate was added and incubated at room temperature for 1.5 hours. Washing for 4 times again, and 200 μL substrate solution was added and the plate was incubated at room temperature for 30 minutes. 50 μL of stop solution was added to each well and the results were read at 450 nm within 30 minutes.

### Pretreatment of PDGF-BB stimulation and inhibitor

To assess the effect of PDGF-BB/PDGFR-β signaling on EPCs induced viability and regenerative capacity of MSCs, cells were pre-treated as follow: (1) MSCs untreated for 24h; (2) MSCs treated with PDGF-BB (Peprotech, USA) for 24 h; (3) co-MSCs untreated for 24h; (4) co-MSCs treated with PDGFR inhibitor AG1296 (TagerMol, USA) for 24 h, respectively.

To assess whether PI3K/Akt and MEK/Erk signaling pathways were involved in PDGF-BB-induced enhancement of MSCs viability, MSCs treated with PDGF-BB were pre-treated for 1 h as follow: (1) untreated for 24 h; (2) treated with PI3K inhibitor LY294002 (MCE, USA); (3) treated with Erk inhibitor PD98059 (MCE, USA); (4) treated with mTOR inhibitor rapamycin (MCE, USA), respectively.

### Real-time PCR

Total RNA was extracted using TRIzol reagent kit (Invitrogen). RNA was reverse transcribed using PrimeScript® RT reagent Kit (TaKaRa). Real-time PCR was performed on an ABI PRISM 7000 sequence detector (Applied Biosystems) using SYBR® Premix Ex Taq™ (Perfect Real Time) (TaKaRa). GAPDH gene was used as internal control. The primers used for analysis were shown in Table 1.

**Table 1:** Primer sequences for real-time PCR used in the study.

**Gene**	**Forward**	**Reverse**
bFGFR	GTCTGCTGACTCCAGTGCAT	CTCCCAGGGGTTTGCCTAAG
CEBPα	TGTATACCCCTGGTGGGAGA	TCATAACTCCGGTCCCTCTG
EGFR	CTGGTTGTGCATTTGCTGTGG	AAAAGTGCCCAACTGCGTGA
FABP4	TGGGCCAGGAATTTGACGAA	GCGAACTTCAGTCCAGGTCA
GAPDH	GTTACCAGGGCTGCCTTCTC	GATGGTGATGGGTTTCCCGT
MAP2	GCGGGTGCATCCAGTTTC	CTCCCAATCAATGCTTCCTCG
Nestin	AGGAAAAGACCATCTGCCCG	GCCTCTCAGCCAGAAACCAT
Osterix	GTAGGACTGTAGGACCGGA	GCCATAGTGAACTTCCTCCTCA
PDGFR-α	CTGGACACTGGGAGATTCGG	CACGGCCTCCAATGATCTCT
PDGFR-β	ATCAGCAGCAAGGACACCAT	GAACGAAGGTGCTGGAGACA
PPARγ	GCAAACCCCTATTCCATGCT	CCACGGAGCTGATCCCAAAG
RUNX2	TGGCAGTCACATGGCAGATT	CTTGGGTGGGTGGAGGATTC
SOX9	GAGGAAGTCGGTGAAGAACGG	CCCTCTCGCTTCAGGTCAG
TUBB3	GGAGGGGCATCTCTTGAGAAC	GCCTCGTTGTAGTAGACGCT

### Cell proliferation assay

Cell proliferation was assessed as following: (1) Cell Counting Kit-8 (CCK-8) assay kit (Beyotime, China): Briefly, cells were seeded in a 96-well cell culture cluster plates, at a density of 2×10^4^ cells per well in 100μL of culture medium. Then 10μL of CCK-8 reagents were added to each well and incubated for 2 hours at 37°C, and the absorbance of the samples was measured at 450nm using an enzyme-linked immunosorbent assay plate reader. (2) Cell cycle analysis: Cells were fixed in 70% ethanol in PBS at -20°C overnight, and then resuspended in PBS containing 40μg/mL PI and 100μg/mL RNAse and incubated for 30 min at 37°C in darkness. Samples were analyzed by flow cytometry. (3) Cell count using the CountBright (Molecular Probes, Eugene, USA): 300μl of cells was stained and 50μl of CountBright absolute counting beads was added. Then cell count was analyzed by flow cytometry.

### *In vitro* multilineage differentiation

Multilineage differentiation potential of MSCs was examined as previously described [15]: (1) Osteogenesis: cells were cultured 21 days in the osteogenic differentiation medium (DMEM-LG containing 10% FBS, 50 μg/ml ascorbate-2 phosphate, 10^-2^ μmol/L dexamethasone, and 10 mmol/L β-glycerophosphate). Osteogenic differentiation was assessed byalizarin red S staining; (2) Adipogenesis: cells were cultured 10 days in the adipogenic differentiation medium (DMEM with 1 g/ml glucose, DMEM-LG) containing 10% FBS, 50 μg/ml of ascorbate-1 phosphate, 0.1 μmol/L dexamethasone and 50 μg/ml indomethacin. Adipogenic differentiation was assessed by oil red O staining; (3) Neurogenesis: cells were cultured in DMEM containing 1.0μmol/L ATRA (Sigma-Aldrich, 10 mmol/L storage concentration in 100% ethanol) for 48h, then washed twice with D-Hank’s and cultured in neural induction medium containing DMEM, 1.6% dimethyl sulfoxide, 160 μmol/L butylated hydroxyanisole, 20 mmol/L KCl, 1.6 mmol/L valproic acid, 8 μmol/L forskolin, 0.8 μmol/L hydrocortisone and 4 μg/mL insulin (all from Sigma-Aldrich) for 72 h. Neurogenic differentiation was assessed by cell morphology and detection of the neural stem cell marker Nestin (Sigma-Aldrich, USA). Briefly, cells were incubated with an anti-nestin antibody at 4ºC overnight, then incubated with DyLight 546-conjugated secondary antibodies (Jackson ImmunoResearch, USA) for 1 h and washed twice with PBS. Nuclei were stained with DAPI (Sigma-Aldrich, USA). The cells were examined under a fluorescence microscope (TE2000-S; Nikon).

### Cell apoptosis detection

Cell apoptosis of MSCs was assessed by detecting phosphatidylserine exposure on the cell plasma membrane with the fluorescent dye from an Annexin V-FITC Apoptosis Detection Kit (KeyGEN Biotech, China) according to the manufacturer’s protocols. Generally, the cells were washed twice with cold PBS, resuspended into a flow tube with 500μL binding buffer at a concentration of 1×10^6^cells/ml. Both 5μL Annexin V-FITC and 5μL propidium iodide were added to the flow tube, then the cells were incubated for 15 minutes in the dark at RT. The samples were analyzed by flow cytometry (BD Bioscience).

### Animal treatment

Sixty 8-week-old male Sprague-Dawley rats (mean weight, 250g) were obtained from Guangdong Medical Laboratory Animal Center and housed in a standard animal facility with 12-h on/off light conditions. All animals were acclimatized for at least one week prior to surgery and allowed free access to standard food and water. The experiments were approved by the Institutional Animal Care and Use Subcommittee of the Third Affiliated Hospital of Sun Yat-sen University. All efforts were made to minimize the suffering of rats during experiments as following: keeping rats alone in a quiet and warm cage, postoperative analgesia with ketoprofen (5mg/kg, subcutaneous injection), wound disinfection, careful administration of respiration 12h postoperatively, feeding with water after anesthesia recovery and normal diet in the 2^nd^ day. When the animal experiments were finished, the rats were sacrificed with inhalation of carbon dioxide.

**Figure 7:**
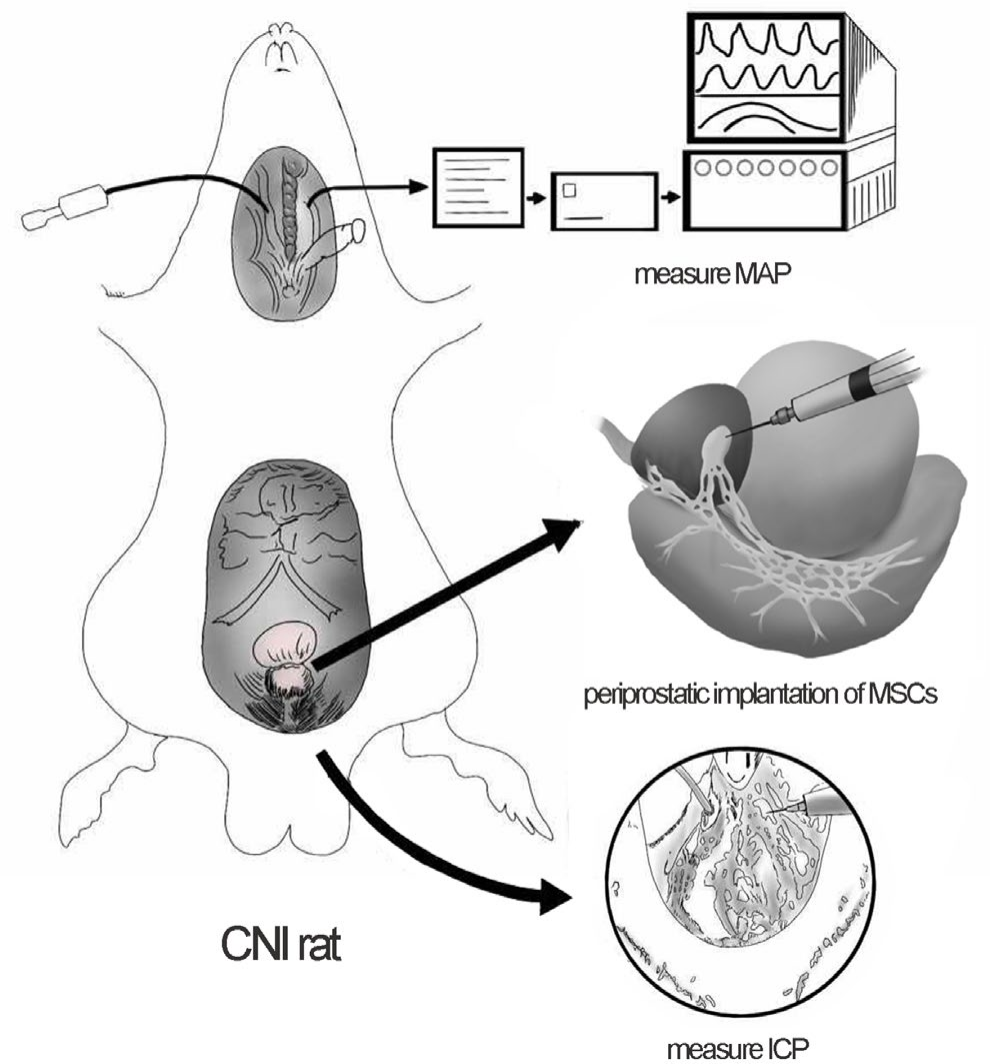
**Periprostatic implantation of MSCs and evaluation of erectile function in CNI rats.** The major pelvic ganglia (MPG) and cavernous nerve (CN) were exposed. Periprostatic implantation of MSCs was applied to restore erectile function of CNI rats. The right carotid artery was exposed to measure the mean arterial pressure (MAP). The corpus cavernosa was exposed and a heparinized 23-gauge butterfly needle was inserted into the penile crus and connected to polyethylene-50 tubing to measure the intracavernous pressure (ICP).

Each rat was intraperitoneally anesthetized with chloral hydrate (0.35ml/100g). A lower abdominal midline incision was used to expose the major pelvic ganglia (MPG) and the cavernous nerve (CN). Bilateral cavernous nerve injury (CNI) was performed in 50 rats (CNI group) and the other 10 rats accepted only laparotomy (sham group). In the CNI group, bilateral CNs were crushed by a non-serrated hemostat (Karl Stortz) as previously described [15]. The hemostat was applied with full tip closure to each CN 1 mm distal to the MPG for 2 minutes. Then the CNI group was randomly divided into five groups, receiving periprostatic implantation of 1*10^6^cells (1) MSCs (M group); (2) MSCs pretreated with PDGF-BB (M+P group); (3) co-MSCs (co-M group); (4) co-MSCs pretreated with PDGFR inhibitor AG1296 (co-M+I group); (5) 20 μL PBS (PBS group). (Figure 7)

### Evaluation of erectile function

Erectile function was evaluated by electrical stimulation of CN as previously described [15]. Briefly, two weeks after treatment, the rats were intraperitoneally anesthetized again and the right carotid artery was exposed to measure the mean arterial pressure (MAP). The corpus cavernosa was exposed and a heparinized 23-gauge butterfly needle was inserted into the penile crus and connected to polyethylene-50 tubing to measure the intracavernous pressure (ICP). The CN was exposed again and stimulated by a bipolar electrode (5 V at 12 Hz for 50 seconds). During tumescence, the maximal ICP (mICP) and total ICP (tICP, area under the curve) were recorded. The ratios of mICP and tICP to MAP were calculated to evaluate the erectile function (Figure 7).

### PKH26 labeling

To determine the *in vivo* viability of MSCs or co-MSCs into the tissues of CNI rats, the cells were stained with PKH26 dye (Sigma-Aldrich, USA) according to the manufacturer’s protocol. PKH26-labeled MSCs were detected in the MPG and cavernous nerve 1, 3, 7 and 14 days after implantation.

### Immunofluorescence staining

The MPG segments were harvested, cut into frozen tissue sections and fixed in methyl alcohol for 10 minutes at 4°C, washed thrice with PBS and blocked with 3% BSA and 0.1% Triton for 1h at room temperature. Then they were incubated with antibodies to S100β (Chemicon; 1:100) at 4°C overnight. Control section was incubated without primary antibody. After washing thrice with PBS, sections were incubated with daylight 488-conjugated secondary antibody (Invitrogen) for 1h and washed thrice with PBS. Nuclei were stained with DAPI. Signals were visualized and digital images were obtained with a fluorescence microscope.

### Masson’s trichrome staining

The penis was harvested, cut into 5μm sections and washed with PBS. The 4% paraform was used for fixation and paraffin for embedding. The tissues were stained with Masson’s trichrome, in which the corpus cavernosum smooth muscle cells appeared red while the collagen fibrils appeared blue. The tissue sections were photographed using a digital camera.

### Western blot

The cells were washed twice with cold PBS and extracted in lysis buffer (Sigma-Aldrich, USA) for 45 minutes on ice. Equal amounts of protein (15–20μg per lane) were electrophoresed on sodium dodecylsulfate polyacrylamide gels (8%), transferred to polyvinylidene fluoride and probed with antibodies as following (All from Cell Signaling Technology, USA; 1:1,000): PDGFR-β, p-PDGFR-β, Akt, p-Akt, mTOR, p-mTOR, p44/42 MAPK (ERK1/2), p-ERK1/2, p70 S6 Kinase, p-p70 S6 Kinase (Thr389 and Ser371). The relative protein expression was normalized to GAPDH.

### Statistical analysis

Statistical analysis was performed with SPSS (version 19.0, USA). All results were expressed as means ± SD. Differences among groups were evaluated using analysis of variance and Newman-Keuls post hoc analysis. All statistical tests were 2-sided, and *P* <0.05 was considered significant.
